# The developmental trajectory of object recognition robustness: Children are like small adults but unlike big deep neural networks

**DOI:** 10.1167/jov.23.7.4

**Published:** 2023-07-06

**Authors:** Lukas S. Huber, Robert Geirhos, Felix A. Wichmann

**Affiliations:** 1 Department of Psychology, University of Bern, Bern, Switzerland; 2 Neural Information Processing Group, University of Tübingen, Tübingen, Germany; 3 Neural Information Processing Group, University of Tübingen, Tübingen, Germany

**Keywords:** object recognition, robustness, out-of-distribution, deep learning, development, generalization, children, deep neural networks, computer vision

## Abstract

In laboratory object recognition tasks based on undistorted photographs, both adult humans and deep neural networks (DNNs) perform close to ceiling. Unlike adults’, whose object recognition performance is robust against a wide range of image distortions, DNNs trained on standard ImageNet (1.3M images) perform poorly on distorted images. However, the last 2 years have seen impressive gains in DNN distortion robustness, predominantly achieved through ever-increasing large-scale datasets—orders of magnitude larger than ImageNet. Although this simple brute-force approach is very effective in achieving human-level robustness in DNNs, it raises the question of whether human robustness, too, is simply due to extensive experience with (distorted) visual input during childhood and beyond. Here we investigate this question by comparing the core object recognition performance of 146 children (aged 4–15 years) against adults and against DNNs. We find, first, that already 4- to 6-year-olds show remarkable robustness to image distortions and outperform DNNs trained on ImageNet. Second, we estimated the number of images children had been exposed to during their lifetime. Compared with various DNNs, children’s high robustness requires relatively little data. Third, when recognizing objects, children—like adults but unlike DNNs—rely heavily on shape but not on texture cues. Together our results suggest that the remarkable robustness to distortions emerges early in the developmental trajectory of human object recognition and is unlikely the result of a mere accumulation of experience with distorted visual input. Even though current DNNs match human performance regarding robustness, they seem to rely on different and more data-hungry strategies to do so.

## Introduction

At a functional level, visual object recognition is at the center of understanding how we think about what we see (Peissig & Tarr, 2007, p. 76). Subjectively, visual object recognition typically seems to be effortless and intuitively easy to us; it is, however, an extremely difficult computational achievement. Arbitrary nuisance variables like object distance (size), pose, and lightning potentially exert a massive influence on the proximal (retinal) stimulus, sometimes resulting in the very same distal stimulus (three-dimensional object in a scene) to have very different proximal stimuli. Conversely, for any given two-dimensional image on the retina—the proximal stimulus—there are an infinite number of potentially very different three-dimensional scenes—distal stimuli—whose projections would have resulted in the very same image (e.g., see DiCarlo & Cox, 2007; Pinto, Cox, & DiCarlo, 2008).^1^ The human ability to recognize objects rapidly and effortlessly across a wide range of identity-preserving transformations has been termed core object recognition (see DiCarlo, Zoccolan, & Rust, 2012, for a review). The computational difficulty notwithstanding, human object recognition ability is not only subjectively effortless, but objectively often tremendously complex (e.g., Biederman, 1987 or see Logothetis & Sheinberg, 1996; Peissig & Tarr, 2007; Gauthier & Tarr, 2015, for reviews).

This computational complexity of (core) visual object recognition is also reflected in the decade-long research efforts it took computational models to reach human-level object classification accuracy. It was not until 2012, when Krizhevsky, Sutskever, and Hinton (2012) trained brain-inspired deep neural networks (DNNs) on 1.3M natural images that computational models began to compete with humans in object recognition tasks. Today, DNNs are the state-of-the-art models in computer vision and surpass human performance on standard object recognition tasks such as image classification on the ImageNet dataset (e.g., see He, Zhang, Ren, & Sun, 2015). It took even longer to obtain models that are not only performing well on natural, undistorted images similar to the training data but also, crucially, on more challenging datasets, so-called out-of-distribution (OOD) datasets containing, for example, image distortions that the models had never seen during training. This task is precisely what humans excel at: robustly recognizing objects even under hitherto unseen viewing conditions and distortions; in machine learning lingo, humans show a high degree of OOD robustness. Even though some of these models have an innovative architecture and/or training procedure—such as CLIP (Radford et al., 2021) or other variants of vision transformers (Dosovitskiy et al., 2021)—the most crucial feature to achieve human-like OOD robustness appears to be training on large-scale datasets (Geirhos et al., 2021). Although standard training on ImageNet includes 1.3M images, most models showing human-like OOD robustness are trained on much larger datasets—ranging from 14M (Big Transfer models; Kolesnikov et al., 2020) to 940M images (semiweakly supervised models; Yalniz, Jégou, Chen, Paluri, & Mahajan, 2019) and even to a staggering 3.6B images (Singh et al., 2022). However, both architecture and data matter—vision transformers, for example, trained on ImageNet, are more robust than standard DNNs trained on ImageNet—but even standard DNNs trained on large-scale datasets (such as Big Transfer models) achieve remarkable robustness. This indicates that large-scale training may be sufficient for OOD robustness in computational models.^2^

It remains an open question, however, whether large-scale experience is also necessary for robust core object recognition. This is precisely the question that we intend to answer with the present study: If large-scale exposure to visual input is indeed necessary to achieve a robust visual representation of objects, then we would expect human OOD robustness to be low in early childhood and to increase with age owing to continued exposure during a lifetime. Alternatively, human OOD robustness might instead result from clever information processing and representation as well as suitable inductive bias (Mitchell, 1980; Griffiths, Chater, Kemp, Perfors, & Tenenbaum, 2010), achieving OOD robustness with comparatively little data. In this case, we would expect human OOD robustness to be already high in early childhood. Both hypotheses can be evaluated with developmental data.

Here we present a detailed investigation of the developmental trajectory of object recognition and its robustness in humans from age 4 to adolescence and beyond. We believe that resolving the competing hypotheses presented may be relevant for understanding crucial aspects of both machine and human object recognition: in terms of machine vision, it is unclear whether large-scale training is the only way to achieve robustness—if children were able to achieve high robustness with little data, this would indicate that the limit of data-efficient robustness has not yet been reached. In terms of human vision, in contrast, the developmental trajectory of object recognition robustness is still a puzzle with many missing pieces, limiting our understanding of the underlying processes and how they develop.

### Development of object recognition

Many cognitive abilities, like language or logical reasoning, mature with time; motor skills, too, take years to develop and be refined. What about our impressive object recognition abilities, particularly robustness to image degradations? Behavioral research investigating the development of object recognition (robustness) in children (after 2 years of age) and adolescents is comparatively sparse, however (for an overview, see the recent preprint by (Ayzenberg & Behrmann, 2022). A number of reviews have pointed out the lack of such studies (Rentschler, Jüttner, Osman, Müller, & Caelli, 2004; Nishimura, Scherf, & Behrmann, 2009; Smith, 2009). Clearly, the ventral visual cortex is subject to structural and functional changes from childhood through adolescence and into adulthood (see Grill-Spector, Golarai, & Gabrieli, 2008; Ratan Murty, Bashivan, Abate, DiCarlo, & Kanwisher, 2021 or Klaver, Marcar, & Martin, 2011, for a review). It has been shown that young children (5–12 years of age) already show adult-like category selectivity for objects in the ventral visual cortex (Scherf, Behrmann, Humphreys, & Luna, 2007; Golarai, Liberman, Yoon, & Grill-Spector, 2010) and that the magnitude of retinotopic signals in V1, V2, V3, V3a, and V4 are approximately the same in children as in adults (Conner, Sharma, Lemieux, & Mendola, 2004). In addition, contrast sensitivity in V1 and V3a also seems to reach adult level by the age of 7 (Ben-Shachar, Dougherty, Deutsch, & Wandell, 2007). These findings indicate that at least neural prerequisites for visual object recognition are in place at a comparatively early age.^3^

Most available behavioral data stem from children younger than 2 years. Already at 6 to 9 months, infants direct their gaze to objects named by their parents, indicating at least a basic form of object recognition (Bergelson & Swingley, 2012, 2015; Bergelson & Aslin, 2017). There are two major developmental changes in those first two years of development. First, children start to use abstract representations of global shape rather than local features to recognize objects (Smith, 2003; Pereira & Smith, 2009; Augustine, Smith, & Jones, 2011). This change enables adult-like performance in simple object recognition tasks and is thought to facilitate generalization and increase the robustness of object recognition (Son, Smith, & Goldstone, 2008). Second, children start to use object shape as the crucial property to generalize names to never before seen objects—a tendency termed shape bias (e.g., see Landau, Smith, & Jones, 1988). An empirical study suggests that these two changes are connected developmentally, such that the ability to form abstract representations of global object shape precedes the shape bias (Yee, Jones, & Smith, 2012). Furthermore, recent work has shown that when recognizing objects, infants (6–12 months) rely on the skeletal structure of objects. That is, the global shape of an object seems to be represented by extracting a skeletal structure (Ayzenberg & Lourenco, 2022).

To our knowledge, only one study has investigated the development of object recognition after the age of 2 systematically. Bova et al. (2007) have shown a progressive improvement of visual object recognition abilities in children from 6 to 11 years of age as measured by a battery of neuropsychological tests.^4^ They report that simple visual abilities (such as shape discrimination) were already mature at the age of 6, whereas more complex abilities (such as the recognition of objects presented in a hard-to-decode way) tended to improve with age.^5^ However, this study did not use stimuli typically used to assess object recognition in adults or DNNs, preventing any quantitative comparisons from being made—something we attempt to remedy with the present study (but see Footnote 17). In the present study, we investigate how well children of different age groups (4–6, 7–9, 10–12, and 13–15) can recognize objects in two-dimensional images at different levels of difficulty (degree of distortions) to trace the developmental trajectory of human object recognition robustness.

## Methods

### General

The methods used in this study are adapted from a series of psychophysical experiments conducted by Geirhos et al. (2018, 2019). The paradigm is an image category identification task to compare human observers and DNNs as fairly as possible. Images are presented on a computer screen, and for each image, observers are asked to choose the corresponding category as quickly and accurately as possible. Concerning the fairness of the comparison between humans and DNNs, one aspect needs to be highlighted: Standard DNNs are typically trained on the ImageNet (ILSVRC) database (Russakovsky et al., 2015), which contains approximately 1.3 million images grouped into 1,000 fine-grained categories (e.g., more than one hundred different dog breeds). However, human observers categorize objects most quickly and naturally at the entry-level, which is very often the basic level, such as, dog rather than German shepherd (Rosch, 1973; Rosch, Mervis, Gray, Johnson, & Boyes-Braem, 1979). To account for this discrepancy and to provide a fair comparison, Geirhos et al. used a mapping from 16 human-friendly entry-level categories to their corresponding ImageNet categories based on the WordNet hierarchy (Miller, 1995).

We adapted the following aspects of the original Geirhos et al. studies to make the paradigm more suitable to test young children: We introduced a certain degree of gamification, added more breaks, and did not force the children to respond within 1,500 ms after stimulus offset to avoid undue stress. After each block of 20 trials, children were free to either quit the experiment or continue with another block. Compared with Geirhos et al., we slightly increased the stimulus presentation duration from 200 ms to 300 ms and only used stimuli that were correctly recognized by at least two adults in the previous studies. We used a between-subject design to test participants on two different types of distortions: binary salt-and-pepper noise and so-called eidolon distortions (Koenderink, Valsecchi, van Doorn, Wagemans, & Gegenfurtner, 2017). In an additional experiment, we used texture–shape cue–conflict stimuli, as in Geirhos et al. (2019). In what follows, we first provide a description of the procedure, the introduced gamification and the employed stimuli. We then proceed by giving details on the tested participants (children, adolescents, adults), the experimental setup, and the evaluated DNNs.

### Procedure

**Figure 1.**
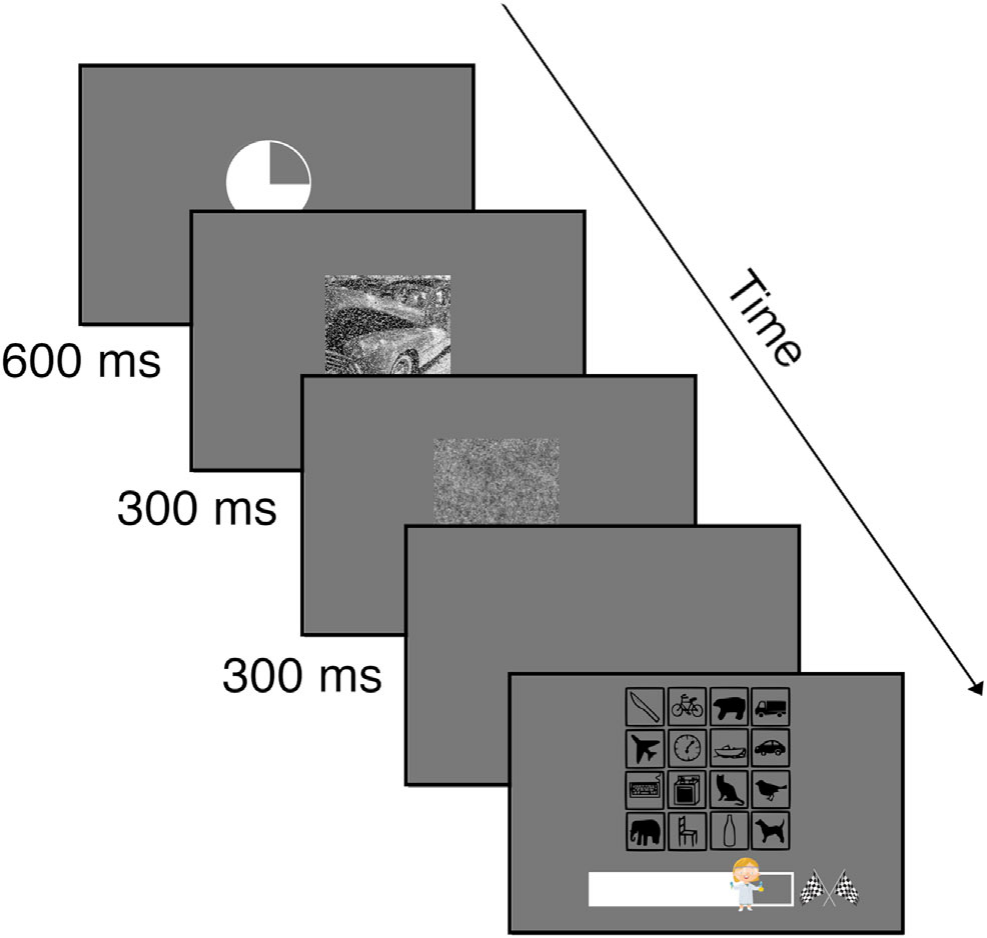
Schematic of a trial. After the attention grabber clock had expired (600 ms), the target image was presented for 300 ms, followed immediately by a full-contrast pink noise mask (1/*f* spectral shape) of the same size and duration. After the mask, participants had unlimited time to indicate their response on the physical response surface. However, participants were instructed to respond as quickly and accurately as possible. After the participant responded, the response surface was shown on the screen, and the experimenter clicked on the icon corresponding with the participant’s response. Icons on the response screen represent the 16 entry-level categories—row-wise from top to bottom: knife, bicycle, bear, truck, airplane, clock, boat, car, keyboard, oven, cat, bird, elephant, chair, bottle, and dog. Below the response surface, there is a gamified progress bar indicating the degree to which the current block has been completed.

Each trial consisted of several phases. First, we presented an attention grabber inspired by an expiring clock (a solid white circle that empties itself within 600 ms) in the center of the screen. We chose a moving stimulus instead of a more commonly used fixation cross to compensate for possible weaker attention in children. Second, the target image was shown in the center of the screen for 300 ms, followed by a full-contrast pink noise mask (1/*f* spectral shape) of the same size and duration to prevent after-images and limit internal processing time. Next, the screen turned blank, and participants were required to indicate their answers. They did this by physically pointing to 1 of 16 icons corresponding with the 16 entry-level categories on a laminated DIN A4 sheet arranged in a 4 × 4 grid (icon size: 3 × 3 cm). We chose this physical response surface mainly for time efficacy (having 4-year-olds handle a computer mouse by themselves can be a lengthy and somewhat unreliable undertaking). Next, the 16 icons appeared on the screen, and the experimenter recorded the response provided by the child using a wireless computer mouse. As in the experiments conducted by Geirhos et al., our icons were a modified version of the ones from the MS COCO website (https://cocodataset.org/#explore). Figure 1 shows the schematic of a trial.

All participants were tested in a separate, quiet room—either in their school (children, adolescents) or at home (adults). The experimental session started with the presentation of example images. For each category, we showed a prototypical example image in the center of the screen and asked participants to name the depicted object. The subsequent presentation of the corresponding category icon indicated the correct category. After completing all 16 examples, participants completed 10 practice trials on undistorted color images (no overlap with stimuli from experimental trials). Extremely rarely, some of the youngest children failed on two or more images and had to complete another round of 10 practice trials. Before the experimental trials started, a single distorted image (matched for the given experimental distortion) was shown, and a short story-like explanation was given to justify why some of the subsequent images would be distorted.^6^ Experimental trials were arranged in blocks containing 20 trials each. After each block, participants received feedback and were asked whether they would like to continue, have a break or terminate the session. Adults were not asked explicitly if they wanted to terminate the session—but of course, all participants were informed at the outset that they could abort the experiment at any given time. Participants could complete a maximum of 16 blocks (320 images) in the eidolon and salt-and-pepper experiments and 20 blocks (400 images) in the cue–conflict experiment.

### Gamification

To increase motivation and make the experiment more appealing to children, we gamified several aspects of the experiment. In the beginning, participants could choose one of four characters (matched for gender) corresponding with four different roles: spy, detective, scientist, or safari guide. The chosen character had to undergo a training session to improve her or his crucial skill. The participants did not know that the crucial skill—identifying objects as quickly and accurately as possible—was the same for all characters. After each trial, the chosen character was displayed at the foremost position of a progress bar indicating how far the participant had progressed in the current block (level). After each block, participants were provided with feedback designed to be perceptually similar to the display of a game score in an arcade game. There were three different types of scores. Participants received 10 coins as a reward for a finished block (not performance related). Additionally, for every two correctly recognized images, they received a star (performance related). If they scored more than eight stars, they earned a special emblem matched for the chosen story character.^7^ Different gamified elements are visualized in Appendix A.

### Stimuli

#### Salt-and-pepper noise and eidolon distortion

**Figure 2.**
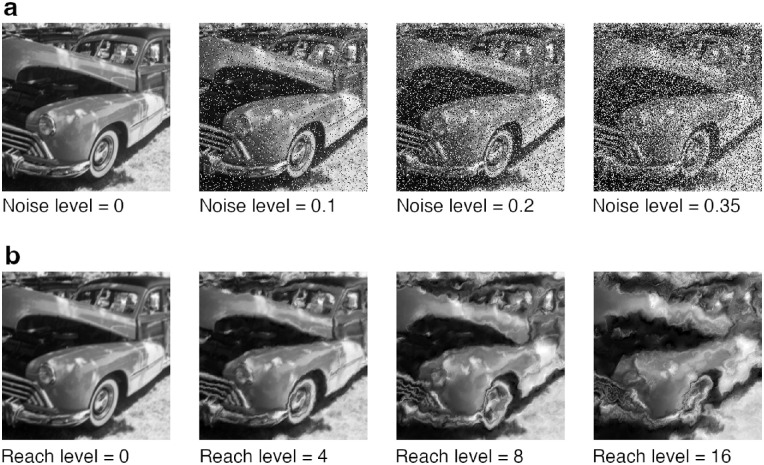
Systematic degradation of images in the salt-and-pepper noise (a) and the eidolon (b) experiments. Note that even though different degradation levels are shown for the same image, participants never encountered the same initial image multiple times.

As mentioned, we used images from 16-class-ImageNet (Geirhos et al., 2018). A subset of 521 stimuli—stimuli that were correctly classified by at least two adults in prior experiments—served as a starting point for the present study. We chose this subset because we feared that children’s motivation might be weaker compared with that of adults and wanted to avoid frustrating children with stimuli that even adults are unable to recognize. We then randomly sampled 320 images (20 for each of the 16 categories) to be manipulated in the next step. For both experiments (eidolon and noise), we manipulated the images to four degrees, resulting in four different difficulty levels per experiment. For the eidolon experiment, we used the eidolon toolbox (Koenderink et al., 2017) with the following settings: grain = 10, coherence = 1 and four different reach levels corresponding with the four difficulty levels (0, 4, 8, and 16). The higher the reach level, the more distorted the images are and the more difficult it is to recognize them. In the noise experiment, a certain proportion of pixels were either set to a gray value of 1 (white) or 0 (black). This manipulation is often referred to as salt and pepper noise. The four difficulty levels in this experiment corresponded with four different proportions of flipped pixels (0, 0.1, 0.2, or 0.35). For example, 0.2 means that 20% of the pixels are switched and 80% remain untouched. For simplicity, we use the term difficulty level to refer to both the different reach levels of the eidolon experiment and the different noise levels of the salt-and-pepper noise experiment. It is important to note, however, that the difficulty levels were not matched precisely between conditions, as can be seen in the results. Figure 2 displays an example image to which both distortions were applied.

For each difficulty level, we randomly selected five images per category to be distorted. Note that in both experiments, the lowest difficulty level (either reach level or proportion of switched pixels equals 0) can be interpreted as only a grayscale transformation of the original color images.^8^ Next, we divided the 320 images into 4 chunks of 80 images each. Such a chunk features 5 images per category and 20 images per difficulty level. From each chunk, we created 4 blocks of 20 images each, resulting in 16 blocks that we later used in the experiment. To minimize predictability, individual blocks of 20 images were not balanced for categories (i.e., a block could contain a variable number of images from a given category). However, each block was balanced for difficulty levels (five images per difficulty level). To keep the participant’s motivation as high as possible, we pseudo-randomized the order of image presentation within each block during the experiment given the following constraint: The first and last three images of a block had to be easy to recognize (difficulty level of one or two).

#### Cue conflict

**Figure 3.**
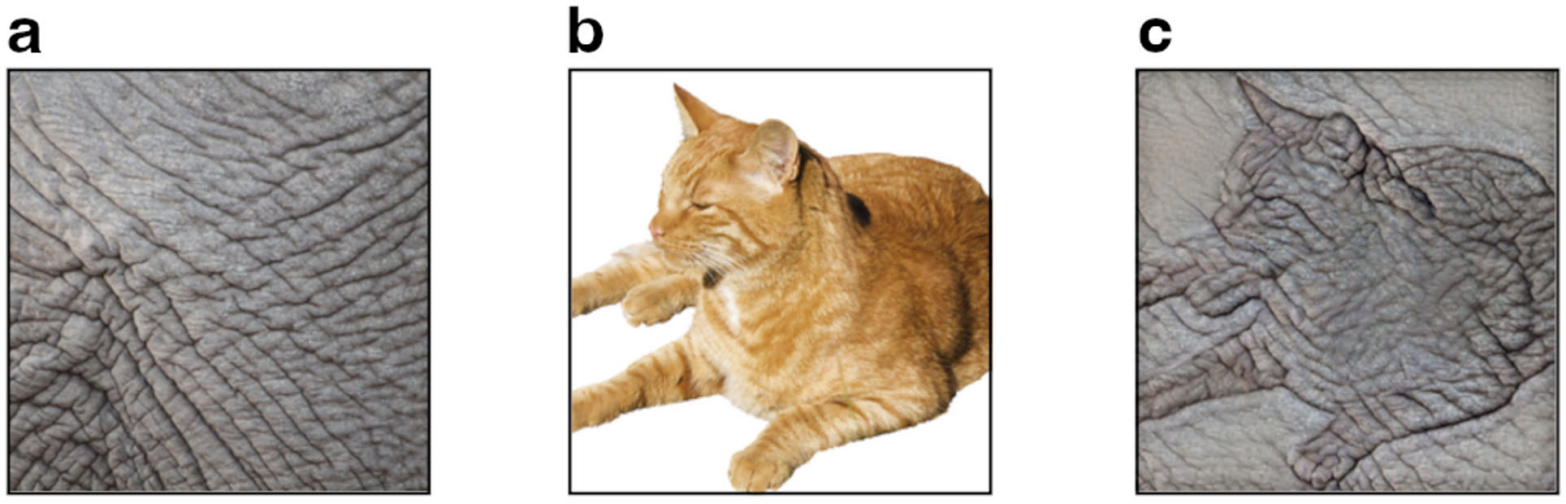
Stimuli generation for the cue conflict experiment. Using style transfer (Gatys et al., 2016), a texture image (a) is combined with a content image (b) to create texture–shape cue conflict stimulus (c). Note that participants never encountered texture and content images. They only encountered texture–shape cue conflict images and original images (similar to content images but featuring natural backgrounds). Figure adapted from Geirhos et al. (2019).

In the cue conflict experiment, we used a subset of images as used in Geirhos et al. (2019). These 224 × 224 pixel cue conflict images are designed to have a conflict between two cues, namely, object shape and object texture, for example, the shape of a cat combined with the texture of elephant skin (see Figure 3). The stimuli were created using the style transfer method (Gatys, Ecker, & Bethge, 2016), whereby the content of an image (shape) is combined with the appearance of another image (texture) using a DNN-based approach. From the 1,280 cue conflict images created by Geirhos et al. (2019), we sampled 240 images (15 per category) to use in this experiment. We included 160 original color images (10 per category) as a baseline to help keep the task intuitive for the children (sampled from the 521-image subset of 16-class-ImageNet as described elsewhere in this article). The whole sample of 400 images was split into 5 chunks of 80 images each—32 original images (2 per category) and 48 cue conflict images (3 per category). As in the other 2 experiments, we created 4 blocks (20 stimuli each) from each chunk, resulting in 20 blocks that we later used in the experiment. The selection of the images was again not balanced regarding categories but for difficulty levels (i.e., each block contained 8 original and 12 cue conflict images). Again, the order of image presentation in the experiment was pseudo-random with the following constraint: The first and last three images had to be original but not cue conflict images.

### Participants

We collected 23,474 trials from a sample of 146 children and adolescents (4–15 years) and 9 adults. Participants were assigned to one of three experiments: Noise (48 children and three adults, 60% female), eidolon (46 children and three adults, 45% female) and cue conflict (52 children and 3 adults, 45% female). Further descriptive information about the sample and observations is presented in Appendix B. We recruited children from 17 different schools in Bern (Switzerland). The adult sample was recruited through personal contacts. All participants reported normal or corrected to normal vision, provided (parental) written consent, and were tested in accordance with national and international norms governing research with human participants. The study was approved by the institutional ethical review board of the University of Bern (no. 2020-08-00003). As a token of appreciation for their participation, children received a book of their choice. Only one child decided to cancel the study right after completing practice trials.

### Apparatus

Programming and stimulus presentation were realized with Python (version 3.8.2) on a Lenovo Thinkpad T490s (Quad-core CPU i5-8365U, Intel UHD 620 graphic card) running Linux Mint 20 Ulyana. We programmed the experiment’s interface with the Psychopy library (Peirce et al., 2019; version 2020.2.4). The 14” screen (356 mm diagonal) had a spatial resolution of 1,920 × 1,200 pixels at a refresh rate of 120 Hz. The measured luminance of the display was 361.4 cd/m^2^, and gamma was set to 2.2. Images were presented at the center of the screen with a size of 256 × 256 pixels, corresponding, at a viewing distance of approximately 60 cm, with 4° × 4° of visual angle.^9^ Note that viewing distance varied somewhat between participants due to children’s agitation. For the whole experiment, the background color was set to a gray value in the [0, 1] range corresponding with the mean grayscale value of all images in the dataset of the particular experiment (eidolon, 0.452; noise, 0.459; and cue conflict, 0.478).^10^ All responses were recorded with a standard wireless computer mouse.

### Models

To investigate the effect of dataset size on model robustness, we selected four representative models from the modelvshuman Python toolbox (Geirhos et al., 2021). The models were chosen according to the following criteria: in terms of training dataset size, they are separated by an approximate log unit each; to a certain degree, they are all derivatives of ResNet building blocks (He et al., 2015); and within the class of models that satisfies the first two constraints, each of them is the very best performing model in terms of OOD accuracy as evaluated on the modelvshuman benchmark—thus they are, as of now, some of the most robust DNNs and, therefore, the strongest DNN competitors for our human to DNN robustness comparison. According to these criteria, the following four models were chosen:

>1M: **ResNeXt**: a ResNeXt-101_32x8d model by Xie, Girshick, Dollár, Tu and He (2017) trained on 1.3M images;
>10M: **BiT-M**: a BiT-M model by Kolesnikov et al. (2020) based on a ResNetV2-152x2 trained on 14M images;
>100M: **SWSL**: a SWSL model by Yalniz et al. (2019) based on a ResNeXt-101_32x16d trained on 940M images;
>1,000M: **SWAG**: a SWAG model by Singh et al. (2022) based on a RegNetY-128GF trained on 3.6B images.

Additionally, we decided to include one widely known DNN, **VGG-19** by Simonyan and Zisserman (2014), for comparison purposes because it is based on a very simple architecture and has been studied extensively in the past.

We used a single feed-forward pass with 224 × 224 pixel RGB images except for the SWAG model, which requires 384 × 384 pixel input. In this case, the images were scaled up to 384 × 384 pixel using PIL.Image.BICUBIC interpolation. For grayscale images (noise and eidolon experiment), all three channels were set to be equal to the grayscale image’s single channel.

## Results

Recent machine learning models have seen tremendous gains in object recognition robustness, predominantly achieved through ever-increasing large-scale datasets. Here we ask whether human robustness, too, may simply result from extensive visual experience acquired during lifetime. If so, we would expect human robustness to be low in young children and to increase over the years. To this end, we performed three comparisons between children of different age groups vs. adults and vs. DNNs. We first investigate the developmental trajectory of object recognition robustness (section on developmental trajectory). Having found essentially adult-level robustness already in young children, we then perform a back-of-the-envelope calculation to estimate bounds on the number of images that children can possibly have been exposed to (section on back-of-the-envelope calculation). Finally, we investigate whether object recognition strategies change over the course of development (section on strategy development).

### Developmental trajectory: Human object recognition robustness develops early

**Figure 4.**
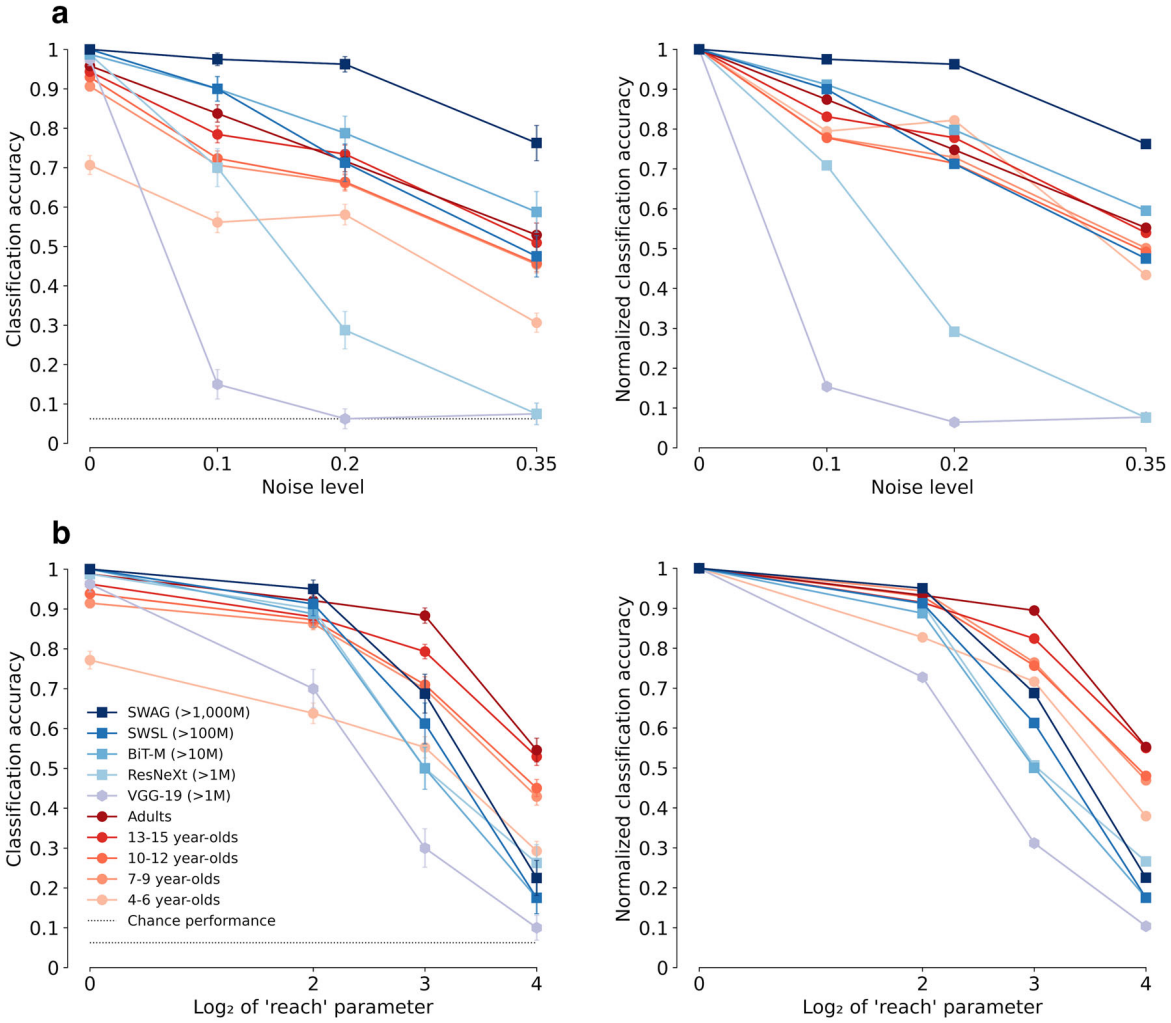
Classification accuracy (top 1) and normalized classification accuracy for different age groups and models. Normalized accuracy shows the change in accuracy relative to the initial accuracy at difficulty level zero of each age group or DNN, respectively. (a) Results for the salt-and-pepper noise. (b) Results for the eidolon experiment. The dotted lines represent chance level performance of 6.25% (100% divided by the number of categories, which was 16). Treating single image classification trials as independent Bernoulli trials, we calculated binomial 95% confidence intervals for all data points using the Wald method (e.g., see Wallis, 2013, and Appendix C for details). Error bars span between the lower and the upper bound of those confidence intervals. Thus, non-overlapping error bars between different observers indicate significant differences in classification accuracy (i.e., that the null hypothesis of zero accuracy difference between the observers is rejected). Additional plots showing the non-binomial standard deviations for the different age groups can be found in Appendix D.

To assess the developmental trajectory of object recognition robustness, we measure classification accuracy depending on the amount of image degradation for two different experiments: salt-and-pepper noise and eidolons (visualized in Figure 2). The results are shown in the left column of Figure 4. In addition to classification accuracy, we plot normalized accuracy with respect to the initial accuracy at difficulty level zero because this makes it easier to disentangle the effects of initial accuracy and change in robustness (right column in Figure 4).

First, looking at classification accuracy, it can be seen that although the adults’ performance is close to ceiling at difficulty level zero, there is a moderate decrease in accuracy as the difficulty level increases (dark red, circles). However, even at difficulty level three, adults still demonstrate a fairly high accuracy far above the chance level. The robustness trajectories for DNNs differ dramatically (shades of blue and violet): older models trained on ImageNet (>1M images) are typically far below the human level (VGG-19; ResNeXt), although modern models trained on large-scale datasets (>10, >100 or even >1,000M images) are sometimes even above the human level, a finding consistent with Geirhos et al. (2021), who reported that the model-to-human gap in OOD distortion robustness has essentially closed. However, the developmental trajectory of robustness during childhood and adolescence has not been studied so far: Across both experiments (salt-and-pepper noise as well as eidolons), overall performance increases as a function of age. The biggest gain in performance is achieved between the groups of 4- to 6-year-olds (light orange, circles) and 7- to 9-year-olds (darker orange, circles). That being said, across all age groups, there seems to be only a linear offset when compared to adults (who have a similar slope): relative to their performance level at zero noise or distortion, even 4- to 6-year-olds seem to have acquired essentially adult-like robustness—i.e., their relative (normalized) performance under noise is similar to that of adults (top right panel) or very nearly so (bottom right panel).

**Figure 5.**
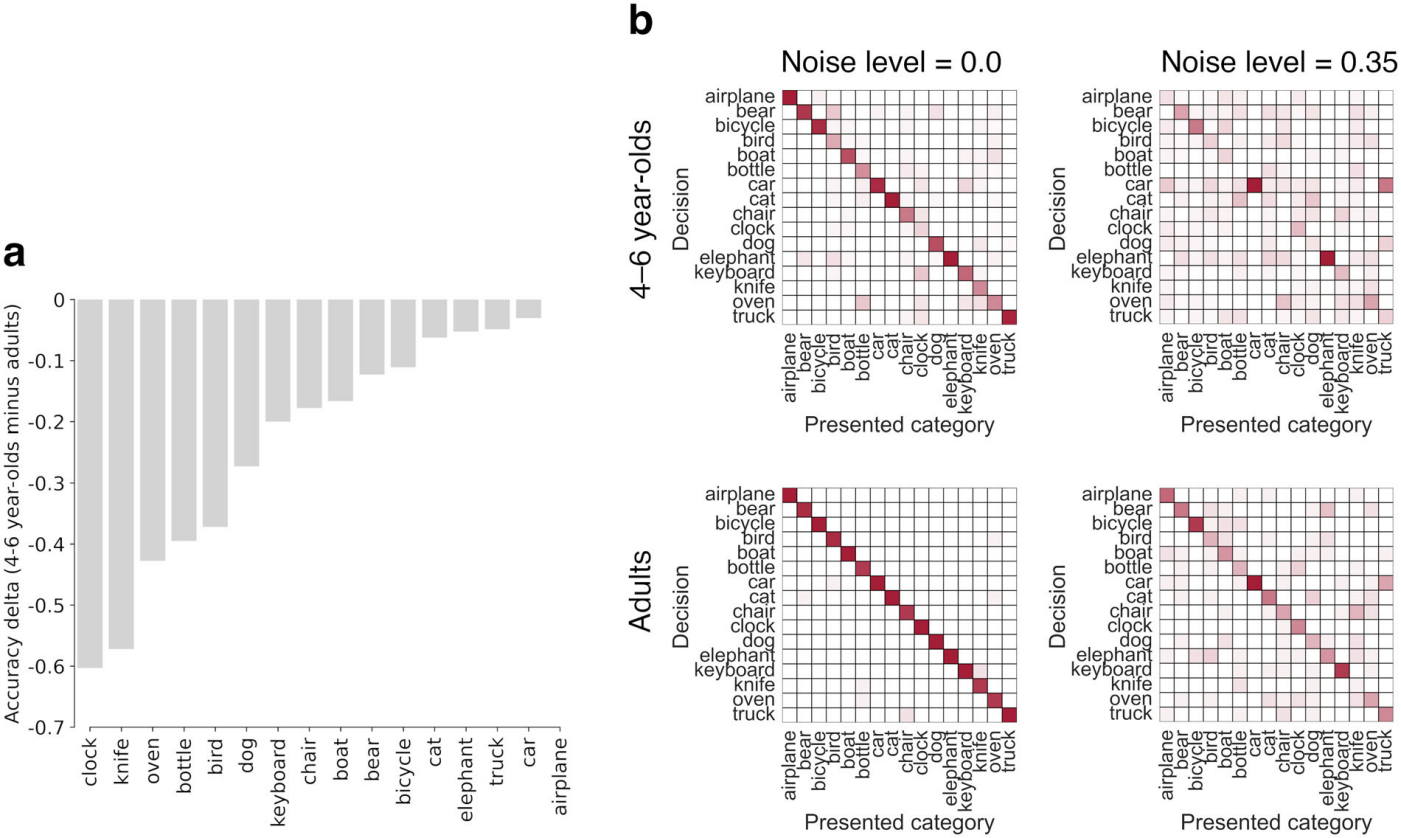
Classification accuracy as a function of different classes. (a) The difference (Delta) of class-wise accuracy between 4- to 6-year-olds and adults on undistorted images (averaged over the salt-and-pepper noise and eidolon experiment). For example, while adults recognized 96.66% of all undistorted clock images, 4- to 6-year-olds only recognized 36.36% correctly—resulting in a Delta of 60.30%. (b) Confusion matrices for 4- to 6-year-olds and adults for undistorted images (noise level = 0.0) and heavily distorted images (noise level = 0.35) in the salt-and-pepper noise experiment. Rows show the classification decisions of observers, and columns show the ground truth label of the presented category. Transparency of single squares within a matrix represents response probabilities (fully transparent = 0%, solid red = 100%). Entries along the negative diagonal represent correct responses; entriesoff the negative diagonal indicate errors.

As shown above, already 4- to 6-year-olds display remarkable levels of object recognition robustness. However, their overall accuracy, even in the noise-free case, is substantially lower than those of older children and adults. Therefore, we ask: Is this difference either due to a generally weaker ability to recognize objects—which indicates a qualitative change in object processing and robustness—or could it be due to a weak performance on a subset of categories, which in turn would indicate only a quantitative change in terms of the number of categories they have already acquired? In Figure 5, we take a closer look at accuracies across different classes. We observe highly nonuniform accuracies: for some classes like airplane, and so on, 4- to 6-year-olds have nearly adult-level accuracy (Subfigure 5a). However, there are also some classes where young children perform substantially worse, such as clock or knife. This overall pattern is confirmed when looking at confusion matrices (Subfigure 5b), which shows that 4- to 6-year-olds maintain high performance on a number of classes, even for severe levels of noise (as indicated by high accuracies (red-ish entries) on the diagonal).^11^ Confusion matrices can be considered as a more fine-grained version of the graphs in Figure 4. In other words, the matrices show how the class-conditional classification accuracies of single object categories change as the distortion increases. The finding that for the 4- to 6-year-olds, the noise-related accuracy decrease does not occur for all categories uniformly, indicating that young children’s weaker overall performance is not due to a generally weaker ability to recognize objects but rather to a weak performance on a subset of categories. Even though 4- to 6-year-olds have not yet acquired robust representations for the same number of categories as adults, they appear almost adult-like regarding some age-appropriate categories they have already acquired. This finding suggests that the change in robustness along the developmental trajectory is rather quantitative (incremental) and not qualitative.

### Back-of-the-envelope calculation: Human robustness does not require seeing billions of images during lifetime

So far, we have seen that robust object recognition emerges early in development and is largely in place by the age of five. After the age of nine, OOD robustness does not seem to increase substantially. This indirectly indicates that for humans—quite different than for DNNS—more data (or experience) does not necessarily imply better robustness. As an attempt to quantify this more directly, and to provide a meaningful comparison with DNNs trained to classify static images, we approximate the accumulated amount of visual experience in human observers by estimating the number of images that those observers are exposed to during their lifetime. We use this estimation to compare different age groups with different models.

We estimated the number of images that human observers are exposed to by calculating the total number of fixations during lifetime for each age group. During a fixation, the eyes remain relatively stationary, and the majority of visual information is received. We thus consider fixations as a good proxy of static input images (e.g., Rucci & Poletti, 2015). To estimate accumulated fixations, we made two assumptions: a) accumulated wake time for any given age group and b) fixations per second for any given age group. Calculating the former is straightforward: During development, wake time gradually increases as a function of age. For example, 0- to 1-year-olds are, on average, awake for 11.5 hours a day, whereas adults are awake for 16.5 hours (Thorleifsdottir, Björnsson, Benediktsdottir, Gislason, & Kristbjarnarson, 2002). We took the mean age of each tested age group and calculated the total accumulated wake time for this particular age in seconds. Estimating the number of fixations per second is more complex: Fixation duration varies to a great extent (100–2000 ms; e.g., see Young & Sheena, 1975; Karsh & Breitenbach, 1983) and is heavily dependent on age and the given visual task (Galley, Betz, & Biniossek, 2015). Thus, as a reference, we chose a task close to an everyday natural setting (a picture inspection task) and for which developmental data are available (Galley et al., 2015). We then calculated fixations per second for each age group based on the fixation duration measured for the mean age of this particular age group. Because there are no available data for adults in the picture inspection task, we estimated the fixation duration of adults by fitting a linear regression line. Fixations per second calculated in this way ranged from 2.56 for 4- to 6-year-olds to 3.42 for adults (mean adult fixation time of 292 ms; see Table F1 in Appendix F for details).

**Figure 6.**
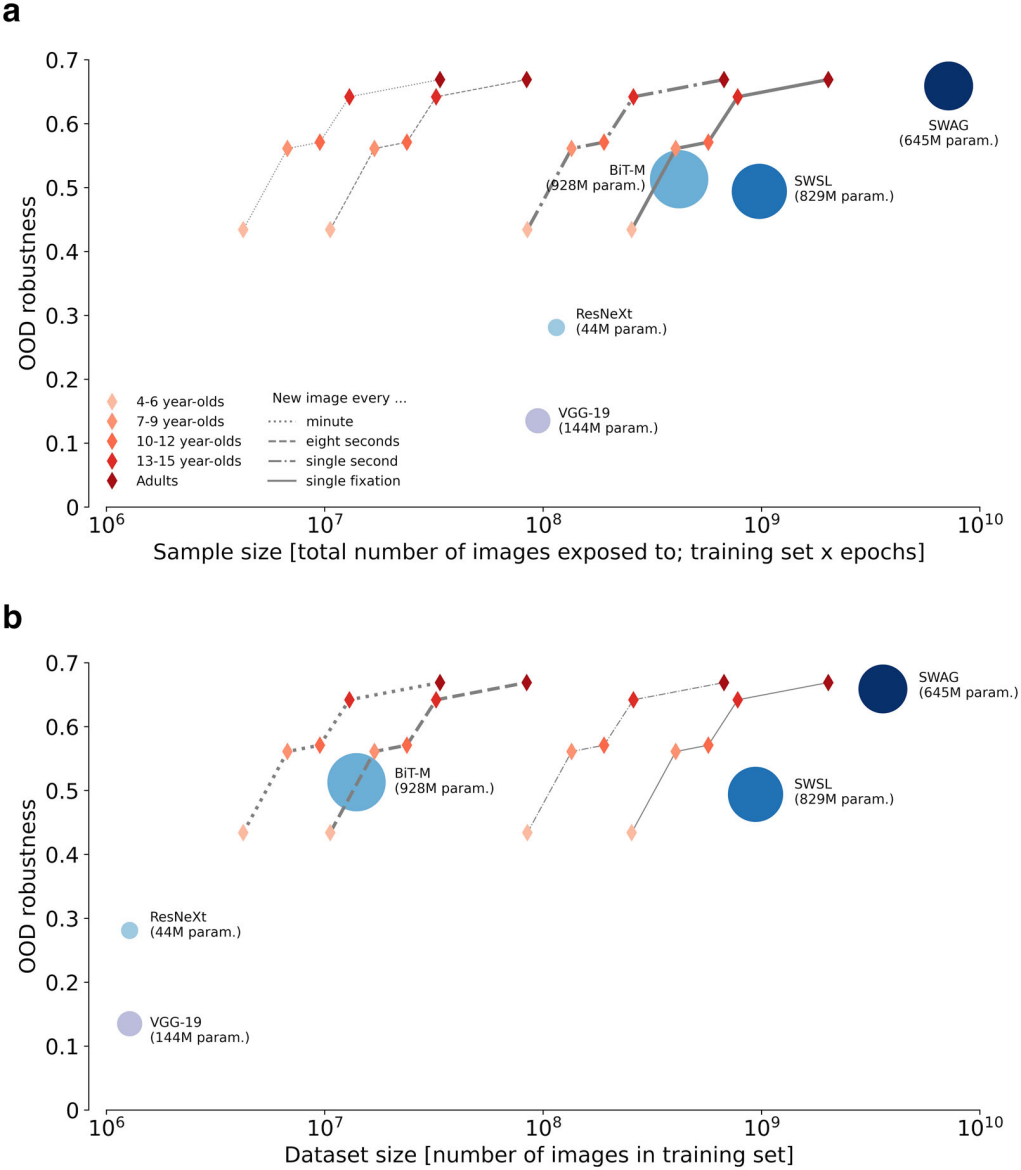
Mean OOD robustness for different age groups and models as a function of (a) sample size and (b) dataset size on semi-logarithmic coordinates. For human observers, four different estimates of the amount of visual input are given (indicated by different line types), resulting in four different trajectories. We suggest that for the comparison regarding sample size, the two most right trajectories, and regarding dataset size, the two most left trajectories should be considered (bold lines). The circle area for models reflects the number of parameters optimised during training.

However, given that visual input does not change significantly for extended periods of time in everyday life, one may not want to count each fixation as a new input image. Furthermore, using head-mounted cameras, it has been shown that frequency distributions of objects in toddlers’ input data are extremely right skewed (Smith, Jayaraman, Clerkin, & Yu, 2018): Toddlers have only experience with very few objects of a specific category, but see those objects (images) very often. It is not clear whether this is just a nonoptimal consequence of the natural learning environment of humans or whether the existence of many similar views of the same object plays an important role in object name learning and thus in learning robust visual representations (Clerkin, Hart, Rehg, Yu, & Smith, 2017). To account for these ambiguities, we provide four different estimates regarding the amount of human visual input. As a minimum, we assume a new image every minute, whereas as a maximum, we assume a new image every single fixation. Additionally, and less extreme, we propose a lower (new image every eight seconds) and an upper (new image every single second) estimate between the bounds set by every minute and every fixation.^12^ Furthermore, there are different choices for counting input images for DNNs. Should every encountered image (sample size = training dataset size × number of epochs, i.e., iterations over the entire training dataset) or the dataset size (number of images in training dataset) be considered as visual input? It is unlikely that training on a smaller dataset for an increased number of epochs yields the same increase in robustness as training on a larger dataset. In fact, the evaluated models vary substantially regarding dataset size (1.28M to 3.6B) and epochs (2 to 90). Thus, we decided to plot human data against both metrics, sample size as well as dataset size (see Table F2 in Appendix F for details on the calculation of input images for DNNs). Figure 6 compares different age groups and models regarding OOD robustness and number of input images for DNNs’ sample size (Subfigure 6a) and dataset size (Subfigure 6b). As a unified measurement of classification robustness, we calculated the mean classification accuracy over all moderately and heavily distorted images (salt-and-pepper noise: noise level 0.2 and 0.35, eidolon: reach levels 8 and 16) for each age group and all models. One important question we had to address was whether to calculate OOD robustness in absolute or relative terms. After careful consideration, we think that there is no correct solution and that there are arguments in favor of both possibilities. One could argue that robustness should be relative to the performance on original undistorted images. An observer that, for example, only gets 50% correct on undistorted images but also gets 50% correct on OOD images would get an OOD robustness score of 1.0. This result would suggest that there is no decrease in accuracy for distorted images relative to the initial accuracy on clean images. To challenge this line of reasoning, one could object that the worse a classifier is overall, the better it would do in terms of OOD robustness if we only looked at relative measures (all the way to the extreme where random guessing achieves perfect OOD accuracy). Based on these considerations, we decided to include both absolute and relative plots in the present paper. Although we show the more conservative absolute plots in Figure 6, the relative plots—suggesting an even more pronounced pattern—are shown in Appendix F.

It is important to note that our back-of-the-envelope calculation is meant to provide a rough estimation and not an exact quantification of the relevant variables. The reported results and plots vary depending on the assumptions made (as explained elsewhere in this article). Furthermore, it could be objected that human and model visual experiences are fundamentally different and cannot be compared per se. One could, for example, argue that while human visual input is continuous, models are only presented with static images.^13^ Additionally, it could be objected that even if visual input could be matched in terms of quantity, there would remain relevant differences in data quality. Whereas models are often trained on random images from the world wide web, humans usually actively choose their fixations such that they are maximally informative (Evans et al., 2011; Callaway, Rangel, & Griffiths, 2021).^14^ Nevertheless, despite these difficulties, we believe that our estimation is reasonable and provides a valid starting point for an important discussion on the data efficiency of humans vs. DNNs with respect to object recognition robustness.

Keeping this in mind, our calculations suggest that human object recognition robustness is more data efficient compared with DNN robustness, irrespective of the choice of metric. For example, focusing on sample size, we find that the two least data-hungry models (ResNeXt and VGG-19) have been exposed to approximately as much input as 4- to 6-year-olds (notably only looking at the two highest of our image number estimates) but are 15% to 30% less robust. The only model comparable with 13- to 15-year-olds in terms of OOD robustness as a function of sample size is SWAG (0.642 vs. 0.659). However, even when counting all fixations as input images—most likely an overestimation of the human external visual input—SWAG needs approximately 10 times more data to achieve human-like OOD robustness (779M vs. 7.2B). A more plausible comparison is probably accomplished by looking at the two more moderate estimates—a new image every single second (dashdotted line) or every 8 s (dashed line)—and comparing them with sample size or dataset size, respectively. Regarding sample size, we find that all three models achieving high OOD robustness (BiT-M, SWSL, and SWAG) need substantially more data than humans to do so. The same is true if we consider dataset size, except for BiT-M, which aligns with the human OOD trajectory (similar OOD robustness of a 6- to 7-year-old and similar dataset size of a 6- to 7-year-old if every awake second is equated with a new image).

It may be important to recall that except for VGG-19, all of the investigated models were chosen since they were the most robust ResNet-based models for a given training dataset size according to the model-vs.-human benchmark (Geirhos et al., 2021). Thus these models represent some of the current best models in terms of data-efficient robustness—comparisons with the many other DNNs would have resulted in even larger discrepancies between humans and DNNs.

Looking only at the different DNNs, we find that BiT-M achieves similar robustness to SWSL with a much smaller dataset (14M vs. 940M). However, this gap almost vanishes when looking at sample size. This indicates that the total number of images exposed to during training (sample size) seems to matter more in terms of OOD robustness than plain dataset size. This may be the case because, owing to data augmentation, images are not exactly the same for every epoch. It has been shown that common data augmentations (such as random crop with flip and resize, color distortion, and Gaussian blur) lead to higher OOD robustness (Perez & Wang, 2017; Mikołajczyk & Grochowski, 2018; Shorten & Khoshgoftaar, 2019). Regarding the number of parameters optimised during training—the area of the circles in the figure—we do not find any direct link to OOD robustness.

### Different strategies: Big models are not like children, but children are like small adults

In the previous sections, we have seen comparisons of overall accuracy and robustness and how this is related to the amount of visual input. While accuracy increases with age (i.e., older children successively categorise more and more images correctly), it remains unclear whether children just gradually acquire more categories, or whether they go through a more radical change of perceptual strategy at some point (at least in terms of overall behavior, teenagers clearly change a lot). To this end, we performed two analyses aimed at understanding how object recognition strategies change (if at all) during childhood and adolescence. The first analysis is related to the image cues used for object recognition (shape or texture), and the second is related to image-level errors (error consistency).

#### Texture–shape cue conflict: No evidence of a strategy change

**Figure 7.**
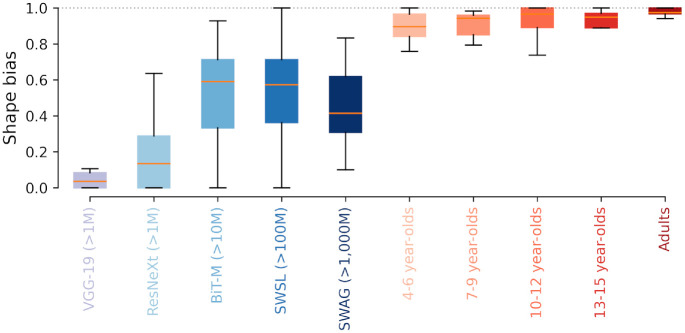
Shape versus texture biases of different models and age groups. Box plots show the category-dependent distribution of shape/texture biases (shape bias: high values, texture bias: low values). For example, 4- to 6-year-olds show a shape bias of 0.88, meaning that of all correct responses, they decided in 88% of cases based on shape cues and in 12% of cases based on texture cues. The dotted line indicates the maximum possible shape bias (100% shape-based decisions). Shape versus texture biases for individual categories are shown in Figure G1 in Appendix G.

Geirhos et al. (2019) and Baker, Lu, Erlikhman, and Kellman (2018) have shown that adults and ImageNet-trained DNNs have a clear discrepancy in object recognition strategy. While human adults base their classification decisions on object shape, DNNs are much more prone to using texture cues instead. To determine whether children are more similar to adults or to DNNs in this regard, we evaluated performance on texture–shape cue conflict images. These images contain conflicting shape and texture information (e.g.,a cat’s shape combined with an elephant’s texture; example stimuli shown in Figure 3). It may be worth pointing out that there is no right or wrong answer in those cases—both the correct texture category and the correct shape category are considered correct responses. Instead, we want to understand whether decisions are consistent with the shape or the texture category. The results are visualized in Figure 7. The exact fractions of shape vs. texture biases and the category wise proportions of texture vs. shape decisions are shown in Appendix G. Those results clearly show that irrespective of age, humans have a very strong shape bias (between approximately 0.88 and 0.97), and there is no evidence to suggest any change of strategy during human development in this regard.^15^ Even models trained on extremely large datasets, however, still do not have a shape bias comparable with humans. In other words, when it comes to using texture or using shape, big models are not like children, but children are like small adults.

#### Error consistency: Distorted input serves as a magnifying glass for object recognition strategies

In the previous section, we have seen that there does not appear to be a radical change in perceptual strategy during childhood when it comes to using texture or shape cues to identify object categories. Nonetheless, all of the previously analysed measures are fairly coarse. Both accuracy and shape bias analyses could potentially overlook more subtle changes of strategy—if, for example, adults struggle with specific images that children find easy, and vice versa, then we would end up with very similar aggregated decisions (as measured by accuracy) despite highly different image-level decisions. Therefore, we here looked at image-level decision patterns through the lens of error consistency (Geirhos, Meding, & Wichmann, 2020). Error consistency is a quantitative analysis for measuring whether two decision-makers systematically make errors on the exact same stimuli. Error consistency between two individual observers is calculated in three steps: First, observed error overlap is calculated by dividing the total number of equal responses—in which both observers either classified an image correctly or incorrectly—by the total number of images both observers have evaluated. Second, because even two completely independent observers with high accuracy will necessarily agree on many trials by chance alone, error overlap expected by chance is calculated (based on the assumption of binomial observers). Third, the empirically observed error overlap is compared against the error overlap expected by chance via Cohen’s κ, which quantifies the agreement of two observers considering the possibility of the agreement occurring by chance.

**Figure 8.**
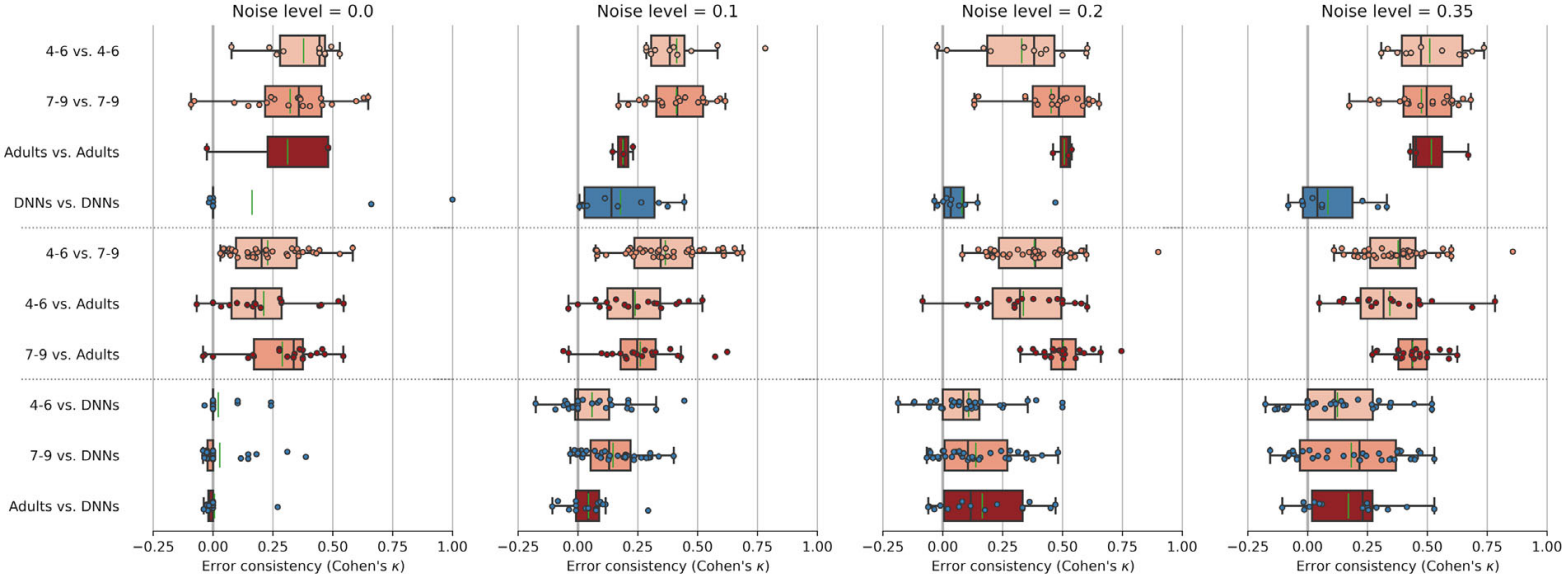
Distorted input serves as a magnifying glass for object recognition strategies—irrespective of age, children make errors on many of the same noisy images as adults; at the same time, models make errors on different images as humans. The plot shows error consistency as measured by Cohen’s kappa (κ) for different distortion levels (columns) split by different within- and between-group comparisons (rows) for a selection of different observer groups (4–6, 7–9, adults, and DNNs). κ = 0 indicates chance level consistency (i.e., both observer groups are using independently different strategies), κ > 0 means consistency above chance level (i.e., both observer groups are using similar strategies), and κ < 0 means inconsistency beyond chance level (i.e., both observer groups use inverse strategies). Plots are horizontally divided into three subsections: Upper subsection (within-group comparisons), middle subsection (between-group comparisons humans only), and lower subsection (between-group comparison humans and DNNs). Colored dots represent error consistency between two single subjects (one of each observer group). Box plots represent the distribution of error consistencies from subjects of the two given observer groups. Boxes indicate the interquartile range (*IQR*) from the first (*Q*1) to the third quartile (*Q*3). Whiskers represent the range from *Q*1 − *IQR* to *Q*3 + *IQR*. While vertical black markers indicate distribution medians, faint vertical green markers indicate distribution means.

Here, we used error consistency to compare four different observer groups (4- to 6-year-olds, 7- to 9-year-olds, adults, and DNNs). We performed all possible within-group (e.g., 4- to 6-year-olds with 4- to 6-year-olds) and between-group (e.g., 4- to 6-year-olds and DNNs) comparisons.^16^ In Figure 8, error consistency is visualized for all difficulty levels of the salt-and-pepper-noise experiment split by different within- and between-group comparisons; the (similar) error consistency plot for the eidolon experiment can be found in Appendix H.

It may be worth pointing out the central patterns: First of all, in line with (Geirhos et al., 2020; Geirhos et al., 2021), human-to-human consistency is generally high, and although it is highest within the same age group, it is also well beyond chance in all between-age-group comparisons. Second, human-to-human error consistency increases as the task becomes harder (i.e., with increasing noise level). Third, regarding comparisons involving DNN models, a very different pattern emerged. Model-to-model consistency starts at chance level and does not increase substantially beyond chance as a function of noise level (highest at noise level 0.1; mean = 0.18). Furthermore, regardless of age, model-to-human consistency is at chance level for noise level zero. And also for distorted images, model-to-human consistency (mean over all model-to-human comparisons for distorted images = 0.127) is far below human-to-human consistency (mean over all human-to-human between-age-group comparisons for distorted images = 0.361). Thus, it almost seems as though distorted input serves as a magnifying glass for object recognition strategies—irrespective of age, children make errors on the same noisy images as adults; at the same time, models make errors on different images as humans.

## Discussion

We investigated the developmental trajectory of core object recognition robustness to assess whether human OOD robustness results from training (experience) on a very large amount of visual input–similar to state-of-the-art OOD-robust DNNs. To this end, we collected 23,474 psychophysical trials from 146 children and 9 adults and compared their OOD performance against five DNNs trained on datasets of different sizes. To our knowledge, this is the first study to directly compare children, adolescents, and different DNNs in a psychophysical core object recognition task using an experimental protocol also employed for adults and in machine learning.^17^

We find that, first, human OOD robustness develops very early and is essentially in place by the age of five. Although there may be a slight increase in robustness as a function of age, by the time children reach middle childhood, they have approximately obtained adult-level robustness (see Figure 4, right column, normalized accuracy). This finding fits with neuroscience data showing that brain maturation relevant for object recognition reaches adult-level at this point of development (Golarai et al., 2010; Scherf et al., 2007; Conner et al., 2004; Ben-Shachar et al., 2007). Furthermore, we find that young children did not perform uniformly weaker on all categories (see Figure 5), indicating that the observed overall improvement in accuracy is due to the acquisition of new categories rather than to a global change in representation and information processing (see Figure 4, left column, accuracy). This allows even 4–6 year-olds to outperform DNNs trained on standard ImageNet. Second, by estimating the visual input for human observers at different points during development, we find that—in contrast to current DNNs—human OOD robustness requires relatively little external visual input (see Figure 6).^18^ This indicates that in humans, OOD robustness may not be achieved solely by the sheer quantity of training data alone. Third, the former two findings are supported by our observations that all tested age groups employ similar object recognition strategies as indicated by a similar shape bias (see Figure 7) and high error consistency across different age groups and difficulty levels (see Figure 8).

Taken together, these findings suggest that for both humans and DNNs, robust visual object recognition is possible but achieved by different means. While human robustness seems fairly data-efficient, at least today, machine robustness is data-hungry.^19^ In other words, there are two different systems with the same property, which came about in different ways—a phenomenon called *convergence* in biology (McGhee, 2011). As an example, consider the ability to fly, which emerged at least three different times during evolution: in mammals (e.g., bats), in sauropsida (e.g., birds) and in insects (e.g., dragonflies). Lonnqvist, Bornet, Doerig, and Herzog (2021) recently argued that considering DNNs and humans as different visual species, and adopting an approach of comparative biology by focusing on the differences rather than the similarities, is a promising way to understand visual object recognition. Accordingly, in what follows, we elaborate on possible differences between human vision and DNN vision, which might explain the difference in data efficiency to solve robust object recognition.^20^

First, there might be a difference in *data quality*, allowing humans to form more robust representations from limited data. While human data is continuous and egocentric (Bambach, Crandall, Smith, & Yu, 2017), this is not the case for standard image databases. Recent advances in data collection using head-mounted cameras allow for developmentally realistic first-person video datasets (Jayaraman, Fausey, & Smith, 2015; Fausey, Jayaraman, & Smith, 2016; Bambach, Crandall, Smith, & Yu, 2018; Sullivan, Mei, Perfors, Wojcik, & Frank, 2020). Although studies have shown that models trained with such biologically plausible datastreams form powerful, high-level representations (Orhan, Gupta, & Lake, 2020) and achieve good neural predictivity for different areas across the ventral visual stream (Zhuang et al., 2021), others find that to match human performance in object recognition tasks models would need millions of years of natural visual experience (Orhan, 2021). A further difference regarding the data quality of humans versus machines lies in the modality of the data; while model input is most often unimodal, human input is multimodal. It has been shown that the availability of information across different sensory systems is linked to the robustness of human perception (e.g., see Ernst & Bülthoff, 2004; Gick & Derrick, 2009; von Kriegstein, 2012; Sumby & Pollack, 1954). Regarding vision, Berkeley (1709) famously argued that “touch educates vision.” Affirmatively, a recent study demonstrated that neural networks trained in a visual-haptic environment (compared with networks trained on visual data only) form representations that are less sensitive to identity-preserving transformations such as variations in viewpoint and orientation (Jacobs & Xu, 2019). Taken together, the continuous, egocentric and multimodal nature of human training data might explain why current DNNs are not as data efficient as humans. Accordingly, a limitation of our study is the lack of systematic variation in the quality of training data. Perhaps by providing DNNs with high-quality training data, even current DNN architectures could achieve OOD robustness with as little data as humans. Thus, future research should systematically acquire multimodal datasets of varying quality and evaluate the trained models on OOD datasets.

Second, humans may rely on different inductive biases—that is, constraints or assumptions prior to training (learning)—allowing for more data-efficient learning. Especially intuitive theories, such as, intuitive physics, theory of mind, or implicit knowledge about the causal structure of the world, might lead to efficient processing of the available data (e.g., see Lake, Ullman, Tenenbaum, & Gershman, 2017; Marcus, 2020; or Goyal & Bengio, 2020) for the role of inductive biases in OOD robustness in general). For example, once learned that the representation of a particular object does change based on certain physical conditions (such as lighting or distance), intuitively knowing that all objects obey the laws of physics and behave in a causally predictable way should facilitate object recognition for other objects which are affected in similar ways. Human inductive biases are the product of millions of years of evolution and are built in right from the start (birth). Thus, to further disentangle the influence of evolution versus lifetime experience, it would be interesting to investigate the developmental trajectory during infancy. In this regard, the present study is limited, however, because the employed experimental set-up does not allow testing children younger than four years of age. We did not test younger children and infants because this would have required us to employ an experimental set-up different to what we used to test adolescents and adults, weakening our comparison. To ensure consistency (also with respect to the DNN comparison), we included no children younger than 4 years of age. However, it is fair to say that 4-year-olds are already quite old by the standards of developmental research.

Third, an exciting possibility is that humans enlarge their initial dataset provided through external input by creatively using already encountered instances to create new instances during offline states—a concept similar to what in reinforcement learning is called experience replay (e.g., see O'Neill, Pleydell-Bouverie, Dupret, & Csicsvari, 2010; Lin, 1991, 1992; Mnih et al., 2015). The idea is that, during imagination and dreaming, stored memories are combined to generate new training data (e.g., see Deperrois, Petrovici, Senn, & Jordan, 2022; Hoel, 2021). Thus, in addition to the external input provided by the sensory system, an internal generative model provides the visual system with additional training data. Putting this into context, one could argue that humans and DNNs might be similar to the extent that they both rely on large-scale datasets to solve object recognition robustness, but are, however, very different in how they attain such large datasets: Although DNNs are entirely dependent on external input, humans are somewhat self-sufficient by producing their own training data from limited external input. Assuming that this hypothesis about the emergence of human OOD robustness is true, the question is whether learning during offline states could make DNNs as data-efficient as humans. The present study only compares how much external input is required to achieve high OOD robustness. Our results are thus not suited to answer this question. However, recently, Deperrois et al. (2022) proposed a model based on generative adversarial networks, which captures the idea of learning during offline states by distinguishing between wake states, where external input is processed, and offline states, where the model is trained by a generative model either by reconstructing perturbed images based on latent representations (similar to simple memory recall as during non-REM sleep) or by generating new visual sensory input based on convex combinations of multiple randomly chosen stored latent representations (similar to the rearranging of stored episodic patterns during REM sleep). Experiments with these models show that introducing such offline states increases robustness and the near separability of latent representations. Further evidence for the benefit of learning during offline states comes from world model–based reinforcement learning. It has been shown that reinforcement learning agents can solve different tasks only by being trained in a latent space which could be conceptually associated with the model’s dreams, imagination or hallucinations (e.g., see Zhu, Zhang, Lee, & Zhang, 2020; Hafner, Lillicrap, Ba, & Norouzi, 2019; Ha & Schmidhuber, 2018).

The three described differences between humans and DNNs might explain the difference in data efficiency found in the present study. However, they are arguably only a small subset of all differences, which might account for the differences in data efficiency. What is clear, however, is that object recognition robustness is not only solvable by a single approach. In evolution, there are often many paths to the same feature. Only by examining the environmental constraints present during phylogenesis can we understand why a particular feature emerged. Not being biological systems, DNNs were not exposed to similar evolutionary pressure as humans, and thus data efficiency seems not to be as crucial as for humans. Accordingly, it comes as no surprise that DNNs are less data efficient than humans. However, the data efficiency of humans seems to be a crucial feature of the human visual system. Thus, to truly understand the robustness of human vision, we need to model not only the behavior (OOD robustness) but also the means by which it is achieved.

## Conclusion

Recent improvements in OOD robustness in machine learning are primarily driven by ever-increasing large datasets, with models trained on several billion images. However, humans achieve remarkable OOD robustness very early in life. Our investigations and calculations suggest that children learn much from relatively little data. Children benefit from accumulating experiences but do not require the same amount of experience as state-of-the-art neural network models, indicating that they are achieving OOD robustness by different means as DNNs. The human visual system appears highly data efficient, which may be an evolutionary advantage. It remains an open question what the sources of this data efficiency are: Is it due to the accumulation of high-quality data alone? High-quality data combined with suitable inductive biases and mechanisms to upcycle data to enlarge the training dataset during offline states such as dreaming? We believe that comparing children with adults and DNNs is a fruitful approach to a better understanding of data efficiency in humans and to perhaps inspire a healthy diet for current data-hungry models without sacrificing their robustness.

## Figures and Tables

**Figure A1.**
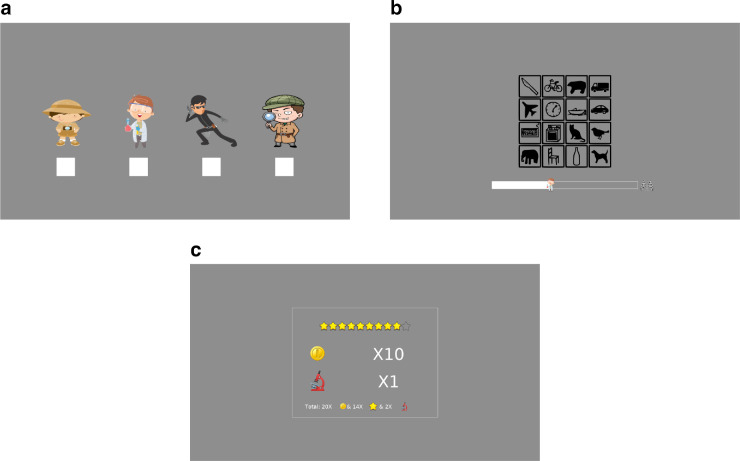
Here we show screenshots at different time-points during the experiment: (a) character selection at the beginning of each session, (b) response screen with gamified progress-bar, and (c) score display with star- and coin-scores and emblem.

**Figure D1.**
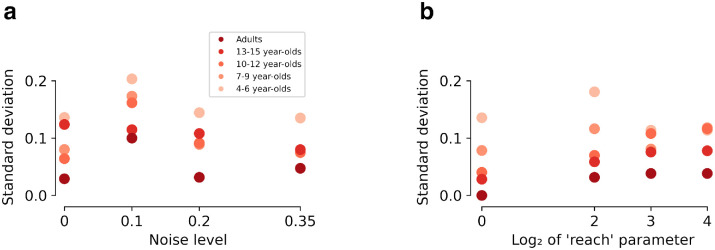
Exploring the variance: Nonbinomial standard deviation for classification accuracies reported in Figure 4. Here we treated each subject’s classification accuracy as a single accuracy measurement. Thus, each data point shows the standard deviation for a set of measurements, which entails the accuracy measurements of all subjects for a particular difficulty level and age group. (a) Results for the salt-and-pepper noise experiment. (b) The same plot for the eidolon experiment. Across both experiments, we observe a tendency for the standard deviation to decrease with age. In other words, we observe that for younger children, there are larger interindividual differences with respect to classification accuracy, independently of the distortion level or type. This indicates that with advancing age, human observers converge in terms of classification accuracy.

**Figure E1.**
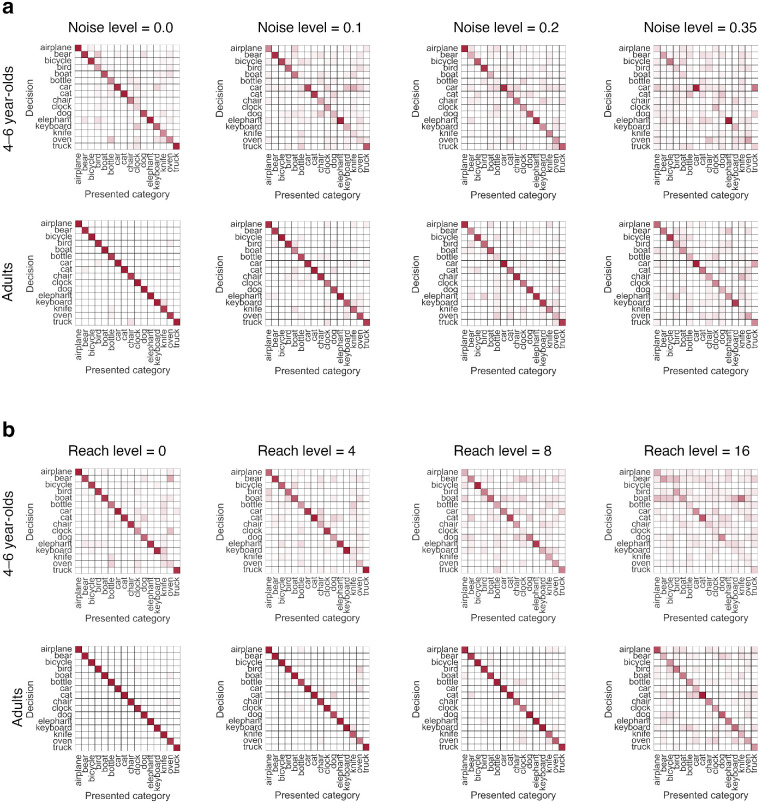
Confusion matrices for 4- to 6-year-olds and adults across all difficulty levels in the salt-and-pepper noise (a) and the eidolon (b) experiment. Rows show the classification decisions of observers and DNNs, and columns show the ground truth label of the presented category. Transparency of single squares within a matrix represents response probabilities (fully transparent = 0%, solid red = 100%). Entries along the negative diagonal represent correct responses; entries off the negative diagonal indicate errors.

**Figure F1.**
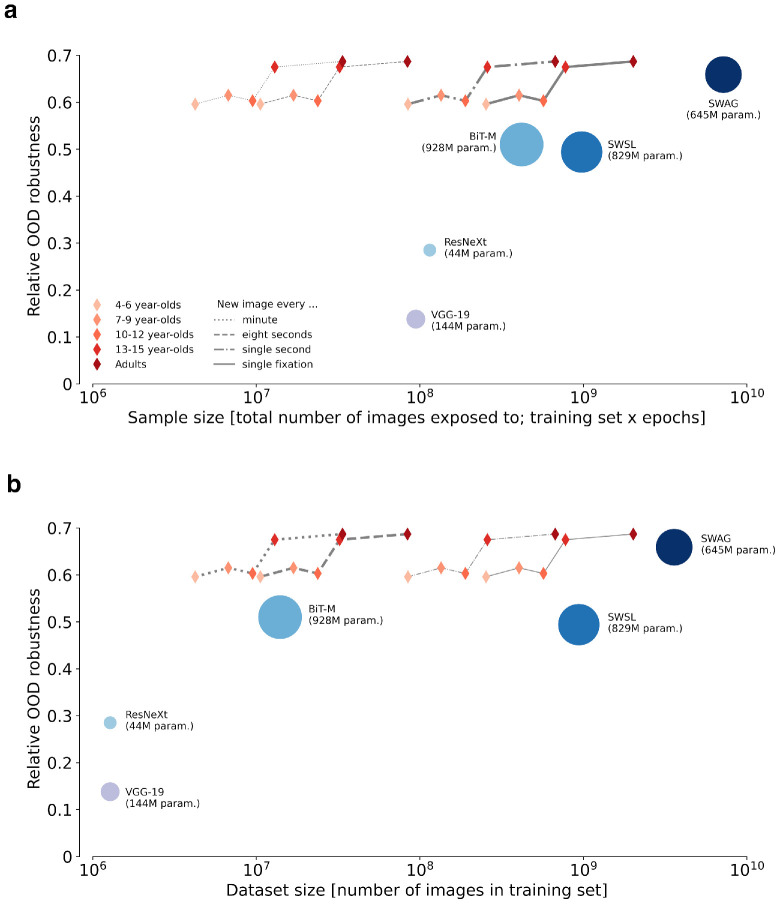
While the plots in Figure 6 show OOD robustness as calculated by the absolute accuracy on moderately and heavily distorted images across experiments, this figure shows OOD robustness calculated as the accuracy on moderately and heavily distorted images relative to the accuracy on clean images. Relative OOD robustness for different age groups and models is shown as a function of (a) sample size and (b) dataset size on semilogarithmic coordinates (x-coordinates of all data points are the same as in Figure 6. For human observers, four different estimates of the amount of visual input are given (indicated by different line types), resulting in four different trajectories. We suggest that for the comparison regarding sample size, the two most right trajectories, and regarding dataset size, the two most left trajectories should be considered (bold lines). The circle area for models reflects the number of parameters optimised during training. The results are similar to Figure 6, but plotting relative OOD robustness reveals even more pronounced differences between the data efficiency of human observers and models.

**Figure G1.**
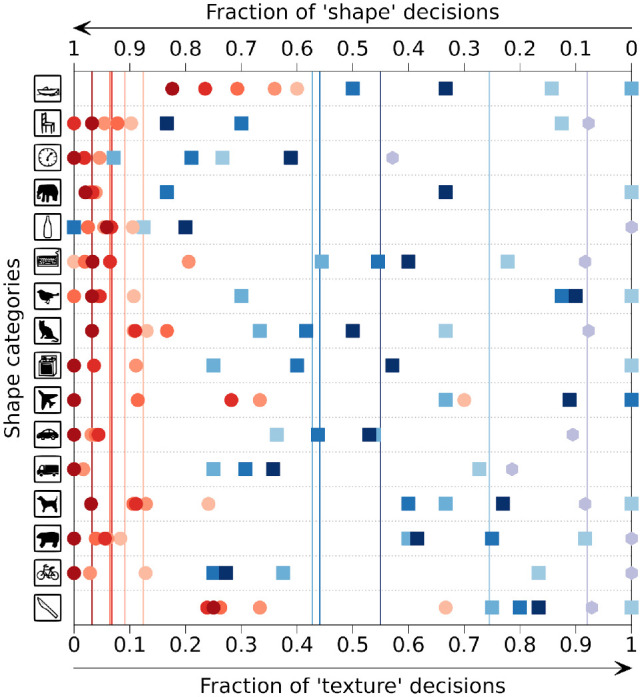
Category wise proportions of texture vs. shape decisions for different age groups (4–6, 7–9, 10–12, 13–15, and Adults) and DNNs (VGG-19, ResNext, BiT-M, SWSL, and SWAG). Only responses corresponding to either the correct texture or correct shape category are considered.

**Figure H1.**
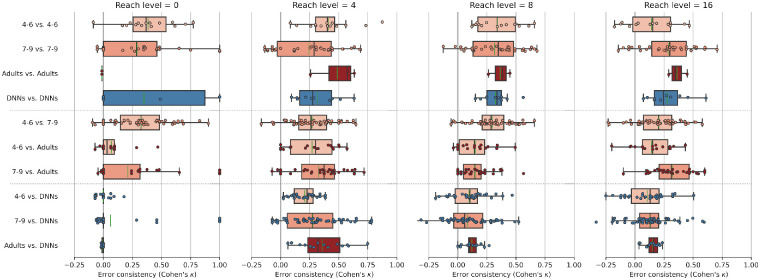
Error consistency as measured by Cohen’s kappa (κ) for different distortion levels (columns) split by different within- and between-group comparisons (rows) for a selection of different observer groups (4–6, 7–9, adults, and DNNs) in the eidolon experiment. κ = 0 indicates chance level consistency (i.e., both observer groups are using independently different strategies), κ > 0 means consistency above chance level (i.e., both observer groups are using similar strategies), and κ < 0 means inconsistency beyond chance level (i.e., both observer groups use inverse strategies). The grid line at κ = 0 is highlighted. Plots are horizontally divided into three subsections: Upper subsection (within-group comparisons), middle subsection (between-group comparisons humans only), and lower subsection (between-group comparison of humans and DNNs). Colored dots represent error consistency between two single subjects (one of each observer group). Box plots represent the distribution of error consistencies from subjects of the two given observer groups. Boxes indicate the interquartile range (*IQR*) from the first (*Q*1) to the third quartile (*Q*3). While vertical black markers indicate distribution medians, vertical green markers indicate distribution means. Whiskers represent the range from *Q*1 − *IQR* to *Q*3 + *IQR*. Compared groups are indicated by the color of dots and boxes, respectively.

## Footnotes

1 At least if the similarity is measured using standard image processing metrics like the Euclidean distance between, or correlation of, the images.

2 It may be possible that large-scale datasets feature (some) images that are similar to images in OOD test sets. This may be particularly likely for common distortions like blur or JPEG compression (Hendrycks & Dietterich, 2019) but considerably less likely for, for example, the eidolon distortions that we use here, which are specifically designed for research purposes.

3 This is not the case for face-selective regions, which continue to develop well into adolescence (Grill-Spector et al., 2008).

4 Tests used included the Efron Test, Warrington’s Figure-Ground Test, the Street Completion Test, the Poppelreuter-Ghent Test, a selection of stimuli from the Birmingham Object Recognition Battery, and a series of color photographs of objects presented from unusual perspectives or illuminated in unusual ways.

5 Note that most tests were very different from the psychophysical task we used in this study. The tests most similar to the task used here are those administered to assess the ability to recognize the structural identity of an object even when its projection on the retina is altered—perceptual categorisation as measured by the Street Completion Test, the Poppelreuter-Ghent Test, and the identification of color photographs of objects viewed from unusual perspectives and presented under unusual lighting conditions. However, all of these tests only consist of a small number of stimuli (11, 13, or 44 respectively), did not apply parameterised distortions to real photographs and did not implement a limited stimulus presentation duration—and thus are not suitable as a rigorous psychophysical assessment of core object recognition.

6 Eidolon: Someone left the images in the rain; that is why some of them are blurry. Noise: Somebody spilled salt and pepper; that is why some of them look a bit strange. Cue Conflict: Someone left the images in the beating sun; that is why some of them stuck together.

7 The emblems were sunglasses for the spy, a magnifying glass for the detective, a microscope for the scientist, and a camera for the safari guide.

8 This is true for the noise images; however, this was not entirely true for the eidolon transformations. As can be seen in Subfigure 3a, the sharpness decreased a little bit compared with the original images—an unforeseen result of the eidolon toolbox.

9 This is only true for the eidolon and noise experiment. Owing to an unnoticed cropping error, the image size in the cue conflict experiment was 224 × 224 pixels, corresponding, at a viewing distance of approximately 60 cm, with only 3.5° × 3.5° of visual angle. We do not think that this small change in absolute size had any influence on the data or results we report.

10 To evaluate the mean grayscale value, images in the cue conflict dataset were converted to grayscale using skimage.color.rgb2gray.

11 Additional confusion matrices can be found in Appendix E.

12 Whereas we argue that the maximum—given by a new image every single fixation—constitutes a naturally occurring constraint, the other three estimates can be considered reasonable guesses.

13 Future studies could consider training models on video data showing objects of categories also featured in the image dataset and then evaluating them on static OOD images. Such an approach would arguably lead to a more adequate comparison with human training data because training data would be better matched between DNNs and humans. However, this lies beyond the scope of the present study.

14 But see Kümmerer, Theis and Bethge (2014) for an investigation of how DNNs—trained on object detection tasks—boost saliency prediction.

15 Note that we use the term shape bias for the tendency to use object shape as the crucial feature to identify an object. However, in the developmental literature, the term shape bias is used to describe the tendency of young children to use object shape as the crucial property to generalize names to objects that were not seen before.

16 Because not all children responded to all stimuli (see Methods section), error consistency sometimes was only calculated on a subset of stimuli. In those cases, we set a minimum constraint of 20 individual stimuli, which had to be evaluated by both observers. Otherwise, error consistency was not calculated.

17 There is one unpublished investigation comparing children and DNNs that was presented at the 20th Annual Meeting of the Vision Sciences Society (Ayzenberg & Lourenco, 2020). They find that young children (4- to 5-year-olds) display remarkable object recognition abilities and outperform a VGG-19 and a ResNet-101 model on perturbed images. Thus, their findings fit our results regarding classification accuracy. However, the current study extends their findings by covering a more extensive age range, using naturalistic images with parameterized distortions and more response categories. Furthermore, we provide a range of additional analyses such as accuracy delta between children and adults, confusion matrices, human–model comparison regarding OOD robustness and input images, texture–shape cue conflict analysis, and error consistency. Additionally, we compare human data with some of the most powerful models to date.

18 Remember, that these results are based on the assumptions that visual experience can be meaningfully quantified by static images and that visual fixations are a good proxy for such static images.

19 Assuming that a) from an evolutionary point of view, robustness is a crucial feature of our visual system (e.g., food detection and predator avoidance under challenging conditions such as in the dark or during snow, rain or fog) and that b) it is not feasible to gather enough visual input during development (even when counting all fixations as new images humans are not gathering as many images as robust DNNs are trained on) to achieve robustness by the same means as modern DNNs (large-scale training), there is a high selection pressure for highly data-efficient learning. In other words, from an evolutionary point of view, our back-of-the-envelope calculation shows that human data-efficient robustness should not be surprising.

20 This elaboration is not thought to be exhaustive but rather to address aspects in which the present study is limited and which we suggest are promising for future research.

## Appendix A: Gamification

In the Appendices, we provide further experimental details as well as supplementary plots and details about the data analysis. Appendix A provides some exemplary screenshots of the visual details of the user interface. Details and characteristics of the tested sample of children and adults are reported in Appendix B. In Appendix C, the reader can find additional details regarding the calculation of the accuracy estimations and the corresponding confidence intervals in Figure 4. Following this, we present additional plots, showing the nonbinomial standard deviations for the different age groups in Appendix D. A full set of confusion matrices for 4- to 6-year-olds and adults for both experiments can be found in Appendix E. Supplementary details regarding the estimation of human input images and dataset and sample size of evaluated models are given in Appendix F, which also features a relative version of the plots in Figure 6. Further, we provide additional details about the results of the cue conflict experiment in Appendix G. Finally, the error consistency plot of the eidolon experiment can be found in Appendix H.

## Appendix B: Demographic characteristics of participants and observations

**Table B1.** Descriptive statistics of participants and observations split by experiments. Sample size and quantity of observations (*n*), as well as mean (*M*) and standard deviation (*SD*) for age and trials within observer groups. Gender distribution (♂/♀) is given in percentages. Note that for adults, trial *M* equals the total number of trials in the respective experiment and trial *SD* is zero because they completed all trials of that particular experiment.

		Age within group				Trials		
Age group	Experiment	*n*	*M*	*SD*	♂/♀	*n*	*M*	*SD*
4- to 6-year-olds	Noise	15	5.13	0.64	33/67	1240	62.66	54.96
	Eidolon	11	5.27	0.65	64/36	1234	102.83	92.06
	Cue-conflict	21	5.29	0.64	62/38	1292	61.52	36.12
7- to 9-year-olds	Noise	9	8.11	0.78	47/53	1840	204.44	112.60
	Eidolon	14	8.36	0.93	43/57	1708	127.14	61.57
	Cue-conflict	11	8.09	0.94	55/45	2020	183.63	118.60
10- to 12-years-olds	Noise	15	11	0.85	47/53	1880	125.33	71.9
	Eidolon	14	11.14	0.77	43/57	1820	130.00	72.64
	Cue-conflict	12	11.08	0.90	50/50	2080	173.33	126.01
13- to 15-years-olds	Noise	9	14.22	0.97	44/56	1280	142.22	82.12
	Eidolon	7	14.00	1.00	71/29	1700	242.86	76.10
	Cue-conflict	8	14.38	0.74	50/50	2260	282.50	107.14
Adults	Noise	3	28.33	5.51	33/67	960	320.00	0.00
	Eidolon	3	26.00	2.65	67/33	960	320.00	0.00
	Cue-conflict	3	29.33	2.89	33/67	1200	400.00	0.00

## Appendix C: Additional details on the calculation of the accuracy estimations and the corresponding confidence intervals in Figure 4

**Table C1.** Continued.

Experiment	Age group	Difficulty	Total trials (*n*)	Total successes (*X*)	Accuracy ($\hat{p}$)	CI bounds
Noise	4- to 6-year-olds	0.0	310	219	0.706	$\hat{p}$ ± 0.024
		0.1	310	174	0.561	$\hat{p}$ ± 0.026
		0.2	310	180	0.581	$\hat{p}$ ± 0.026
		→ 0.35	310	95	0.306	$\hat{p}$ ± 0.024
	7- to 9-year-olds	0.0	460	417	0.907	$\hat{p}$ ± 0.012
		0.1	460	325	0.707	$\hat{p}$ ± 0.020
		0.2	460	304	0.661	$\hat{p}$ ± 0.020
		0.35	460	209	0.454	$\hat{p}$ ± 0.021
	10- to 12-year-olds	0.0	470	437	0.930	$\hat{p}$ ± 0.011
		0.1	470	340	0.723	$\hat{p}$ ± 0.019
		0.2	470	312	0.664	$\hat{p}$ ± 0.020
		0.35	470	215	0.457	$\hat{p}$ ± 0.021
	13- to 15-year-olds	0.0	320	302	0.944	$\hat{p}$ ± 0.012
		0.1	320	251	0.784	$\hat{p}$ ± 0.021
		0.2	320	235	0.734	$\hat{p}$ ± 0.023
		0.35	320	163	0.509	$\hat{p}$ ± 0.026
	Adults	0.0	240	230	0.958	$\hat{p}$ ± 0.012
		0.1	240	201	0.838	$\hat{p}$ ± 0.022
		0.2	240	172	0.717	$\hat{p}$ ± 0.027
		0.35	240	127	0.529	$\hat{p}$ ± 0.030
	VGG-19	0.0	80	78	0.975	$\hat{p}$ ± 0.016
		0.1	80	12	0.150	$\hat{p}$ ± 0.037
		0.2	80	5	0.063	$\hat{p}$ ± 0.025
		0.35	80	6	0.075	$\hat{p}$ ± 0.027
	ResNeXt	0.0	80	79	0.988	$\hat{p}$ ± 0.011
		0.1	80	56	0.700	$\hat{p}$ ± 0.048
		0.2	80	23	0.288	$\hat{p}$ ± 0.048
		→ 0.35	80	6	0.075	$\hat{p}$ ± 0.027
	BiT-M	0.0	80	79	0.988	$\hat{p}$ ± 0.011
		0.1	80	72	0.900	$\hat{p}$ ± 0.031
		0.2	80	63	0.788	$\hat{p}$ ± 0.043
		0.35	80	47	0.588	$\hat{p}$ ± 0.052
	SWSL	0.0	80	80	1.000	$\hat{p}$ ± 0.000
		0.1	80	72	0.900	$\hat{p}$ ± 0.031
		0.2	80	57	0.713	$\hat{p}$ ± 0.048
		0.35	80	38	0.475	$\hat{p}$ ± 0.053
	SWAG	0.0	80	80	1.000	$\hat{p}$ ± 0.000
		0.1	80	78	0.975	$\hat{p}$ ± 0.016
		0.2	80	77	0.963	$\hat{p}$ ± 0.020
		0.35	80	61	0.763	$\hat{p}$ ± 0.045
Eidolon	4- to 6-year-olds	0	302	233	0.772	$\hat{p}$ ± 0.022
		4	304	194	0.638	$\hat{p}$ ± 0.026
		8	304	168	0.553	$\hat{p}$ ± 0.027
		16	304	89	0.293	$\hat{p}$ ± 0.024
	7- to 9-year-olds	0	445	407	0.915	$\hat{p}$ ± 0.012
		4	445	384	0.863	$\hat{p}$ ± 0.015
		8	445	311	0.699	$\hat{p}$ ± 0.020
		16	445	191	0.429	$\hat{p}$ ± 0.022

**Table C1.** Here, we show the exact quantities involved in the calculation of the accuracy measurements and the corresponding binomial 95-percent confidence intervals in Figure 4. Each psychophysical measurement (trial) that constitutes a data point (classification accuracy of a given age group or model at a certain distortion level) is treated as an independent Bernoulli trial such that a “success” corresponds to a correct classification and a “failure” to a misclassification. Accordingly, we assume that the number of “successes” is a random variable *X* following a binomial distribution B(n,p), whereby *n* is the number of observations and *p* is the probability of “success”. We estimate *p* by the sample proportion $\hat{p}=X/n$, which is essentially the reported accuracy given by dividing the number of correctly classified images by the total number of trials. We calculate the binomial 95-percent confidence intervals for each data point by the following formula (Wald method):
$$\begin{array}{c} \hat{p}\pm \sqrt[{Z}]{\frac{\hat{p}\left(1-\hat{p}\right)}{n}}, \end{array}$$whereby *z* is the quantile of a standard normal distribution corresponding to the target error rate α. For a standard two-tailed 95-percent confidence interval, α = 0.025 and thus *z* = 1.96. To give an example (row indicated by an arrow), consider the classification accuracy of 4- to 6-year-olds on salt-and-pepper noise images (*Difficulty* =0.35). Out of 310 collected trials (*n)*, 4–6 year-olds classified 95 images correctly (*X*). This allows us to calculate the classification accuracy (sample proportion) by dividing the number of correctly classified images by the total number of trials: $\hat{p}=95/310=0.306$. We then use $\hat{p}$ as an estimator of the true population accuracy *p* such that X∼B(310,0.306). According to the above formula, binomial confidence intervals can now be calculated by
$$\begin{array}{c} 0.31\pm \sqrt[{1.96}]{\frac{0.306(1-0.306)}{310}}, \end{array}$$resulting in a 95% confidence interval of [0.282, 0.330] around the mean accuracy of 0.306. Pairwise comparing confidence intervals between different observers allows for determining whether the two corresponding classification accuracy estimations differ significantly. For example, the classification accuracy of the ResNeXt model (row indicated by an arrow) for heavily distorted salt-and-pepper noise images is 0.075 with a confidence interval of [0.048, 0.102]. Since the confidence intervals [0.282, 0.330] and [0.048, 0.102] do not overlap, we conclude that the classification accuracy differs significantly between 4- to 6-year-olds and the ResNeXt model.

Experiment	Age group	Difficulty	Total trials (*n*)	Total successes (*X*)	Accuracy ($\hat{p}$)	CI bounds
	10- to 12-year-olds	0	455	427	0.938	$\hat{p}$ ± 0.010
		4	455	397	0.873	$\hat{p}$ ± 0.014
		8	455	323	0.710	$\hat{p}$ ± 0.020
		16	455	205	0.451	$\hat{p}$ ± 0.022
	13- to 15-year-olds	0	425	409	0.962	$\hat{p}$ ± 0.008
		4	425	374	0.880	$\hat{p}$ ± 0.014
		8	425	337	0.793	$\hat{p}$ ± 0.018
		16	425	225	0.529	$\hat{p}$ ± 0.022
	Adults	0	240	237	0.988	$\hat{p}$ ± 0.006
		4	240	221	0.921	$\hat{p}$ ± 0.016
		8	240	212	0.883	$\hat{p}$ ± 0.019
		16	240	131	0.546	$\hat{p}$ ± 0.030
	VGG-19	0	80	77	0.963	$\hat{p}$ ± 0.020
		4	80	56	0.700	$\hat{p}$ ± 0.048
		8	80	24	0.300	$\hat{p}$ ± 0.048
		16	80	8	0.100	$\hat{p}$ ± 0.031
	ResNeXt	0	80	79	0.988	$\hat{p}$ ± 0.011
		4	80	72	0.900	$\hat{p}$ ± 0.031
		8	80	40	0.500	$\hat{p}$ ± 0.053
		16	80	21	0.263	$\hat{p}$ ± 0.046
	BiT-M	0	80	80	1.000	$\hat{p}$ ± 0.000
		4	80	71	0.888	$\hat{p}$ ± 0.033
		8	80	40	0.500	$\hat{p}$ ± 0.053
		16	80	14	0.175	$\hat{p}$ ± 0.040
	SWSL	0	80	80	1.000	$\hat{p}$ ± 0.000
		4	80	73	0.913	$\hat{p}$ ± 0.029
		8	80	49	0.613	$\hat{p}$ ± 0.051
		16	80	14	0.175	$\hat{p}$ ± 0.040
	SWAG	0	80	80	1.000	$\hat{p}$ ± 0.000
		4	80	76	0.950	$\hat{p}$ ± 0.023
		8	80	55	0.688	$\hat{p}$ ± 0.049
		16	80	18	0.225	$\hat{p}$ ± 0.044

## Appendix F: Back-of-the-envelope calculation

**Table F1.** Details regarding the estimation of the number of input images for human observers. The estimate of accumulated Wake time (in millions of seconds) is based on (Thorleifsdottir et al., 2002). Fixation Duration (in milliseconds) refers to the fixation duration in a picture inspection task (Galley et al., 2015) and is used to calculate *Fixations per second*. Because there was no available data regarding the fixation duration of adults in this task, we assumed a linear relationship between age and fixation duration and used the children’s data to fit a simple regression model to estimate the fixation duration of adults ($\hat{y}=-4.33X+413.66$). Plugging in the mean age of adults (*M* = 28) yields a predicted fixation duration of 292.42 for adults. Min (in millions) refers to the minimal assumed number of input images—a new image every minute. Max (in millions) refers to the maximal possible number of input images—a new image every single fixation. Lower and Upper (in millions) refers to a—what we believe—reasonable estimate of the lower and upper bound of input images encountered during lifetime. The lower bound is calculated by scaling the total number of fixations by 24 (new images approximately every eight seconds) and the upper bound by 3 (new images approximately every single second). E.g., at the age of five, a child has been awake for approximately 99.41 million seconds; it has made about 254.5 million fixations during this time (99.41 × 2.56). Based on these numbers, we estimate that a five year-old child has most likely not seen less than 10.6 and not more than 84.83 million images (total number of fixations during lifetime either scaled down by a factor of 24 or 3).

Age group	*M* age	Wake time	Fixation duration	Fixations per second	Min	Lower	Upper	Max
4–6 year-olds	5.13	99.41	390.00	2.56	4.24	10.60	84.83	254.49
7–9 year-olds	8.11	154.16	380.00	2.63	6.76	16.90	135.15	405.44
10–12 year-olds	11.00	211.54	370.00	2.70	9.52	23.80	190.39	571.16
13–15 year-olds	14.22	272.42	350.00	2.86	12.99	32.43	259.43	779.12
Adults	28.33	591.71	292.42	3.42	33.73	84.32	674.55	2023.65

**Table F2.** Model details regarding all employed models. *Dataset size* refers to the number of images in the training set and is plotted on the x-axis in Subfigure 6b. The sample size equals the number of encountered images during training (dataset size × epochs) and is plotted on the x-axis in Subfigure 6a. *Note that the SWSL model was trained one epoch on 940M images and then 30 epochs on standard ImageNet (1.28M images)—thus, sample size equals 940*M* + 30 × 1.28*M*. Parameters refer to the total number of parameters optimised during training and is represented by the area of the circles throughout Figure 6.

Model	Dataset size	Epochs	Sample size	Parameters
VGG-19	1.28M	74	94.72M	144.00M
ResNeXt	1.28M	90	115.20M	44.00M
BiT-M	14.00M	30	420.00M	928.00M
SWSL	940.00M	1+30*	978.40M	829.00M
SWAG	3.60B	2	7.2B	644.80M

## Appendix G: Shape-bias

**Table G1.** Exact fractions of texture vs. shape decisions of different age groups and DNNs in percent.

Observer	Shape bias	Texture bias
4–6 year-olds	87.55	12.45
7- to 9-year-olds	90.86	9.14
10- to 12-year-olds	93.52	6.48
13- to 15-year-olds	93.18	6.82
Adults	96.72	3.28

VGG-19 (>1M)	7.96	92.04
ResNeXt (>1M)	25.52	74.48
BiT-M (>10M)	57.24	42.76
SWSL (>100M)	55.88	44.12
SWAG (>1,000M)	45.00	65.00
